# Genetic Variation and the Distribution of Variant Types in the Horse

**DOI:** 10.3389/fgene.2021.758366

**Published:** 2021-12-02

**Authors:** S. A. Durward-Akhurst, R. J. Schaefer, B. Grantham, W. K. Carey, J. R. Mickelson, M. E. McCue

**Affiliations:** ^1^ Department of Veterinary Population Medicine, University of Minnesota, Minneapolis, MN, United States; ^2^ Interval Bio LLC, Mountain View, CA, United States; ^3^ Department of Veterinary and Biomedical Sciences, University of Minnesota, Minneapolis, MN, United States

**Keywords:** genetic variation, whole genome sequence, variant discovery, equine, breed differences, genetics

## Abstract

Genetic variation is a key contributor to health and disease. Understanding the link between an individual’s genotype and the corresponding phenotype is a major goal of medical genetics. Whole genome sequencing (WGS) within and across populations enables highly efficient variant discovery and elucidation of the molecular nature of virtually all genetic variation. Here, we report the largest catalog of genetic variation for the horse, a species of importance as a model for human athletic and performance related traits, using WGS of 534 horses. We show the extent of agreement between two commonly used variant callers. In data from ten target breeds that represent major breed clusters in the domestic horse, we demonstrate the distribution of variants, their allele frequencies across breeds, and identify variants that are unique to a single breed. We investigate variants with no homozygotes that may be potential embryonic lethal variants, as well as variants present in all individuals that likely represent regions of the genome with errors, poor annotation or where the reference genome carries a variant. Finally, we show regions of the genome that have higher or lower levels of genetic variation compared to the genome average. This catalog can be used for variant prioritization for important equine diseases and traits, and to provide key information about regions of the genome where the assembly and/or annotation need to be improved.

## 1 Introduction

Genetic variation is a key contributor to health and disease, and understanding the link between an individual’s genotype and the corresponding phenotype is a major goal of genetic research (Genomes Project et al., 2010). Whole genome sequencing (WGS) within and across populations enables highly efficient variant discovery and elucidation of the molecular nature of virtually all genetic variation, from single nucleotide polymorphisms (SNPs) to copy number variants (CNVs) and other large structural variants (Sudmant et al., 2015). Large-scale studies of genetic variation in humans have dramatically improved our understanding of genetic variation across a species and within populations (Genomes Project et al., 2012). Large-scale variant catalogs establish patterns of variation across the genome, including non-coding regions (Mu et al., 2011), permitting elucidation of regional variability in mutation and recombination rates. Knowledge of the background genetic “noise” helps to decipher the link between genotype and phenotype—allowing filtering of likely neutral variants from potentially deleterious variants in genomic regions of interest or within biologic candidate genes.

The equine reference genome (Wade et al., 2009) has provided a key basis for genetic investigations in horse populations (Rebolledo-Mendez et al., 2015; Raudsepp et al., 2019). However, despite consistent progress in our understanding of genetic disease in the horse, disease-causing variants have been identified for less than 20% of the currently recognized genetic diseases (Online Mendelian Inheritance in Animals, OMIA). Similar to humans, many of the significant GWAS regions of interest found for equine traits have been in non-coding regions of the genome. The unknown function of these regions has been a barrier to pinpointing and confirming the causal variant, which is necessary to develop effective genetic tests or targeted treatments. A more complete account of “normal” genetic variation in the horse is critical to establishing the link between genotype and phenotype (Yngvadottir et al., 2009; Genomes Project et al., 2010).

Here we provide the first large-scale catalog of genetic variation in the horse derived from WGS, with the intent of describing variant numbers, types, allele frequencies, and genomic location distribution across the general population and within and across major breeds.

## 2 Materials and Methods

### 2.1 Samples

Paired-end whole genome sequencing was performed on 534 horses using Illumina technology (Supplementary Table S1). Forty-four different breeds predominantly from North America and Central Europe were selected (Supplementary Table S2) based on availability of Illumina WGS from previous and ongoing studies (Finno et al., 2015; McCoy et al., 2016; Norton et al., 2016, 2019; Schultz 2016; Bellone et al., 2017; Schaefer et al., 2017), publicly available data from the Sequence Read Archive (Leinonen et al., 2011), and with the aim of collecting a minimum of 15 individuals per breed for 10 target breeds (Arabian, Belgian, Clydesdale, Icelandic, Morgan, Quarter Horse, Shetland, Standardbred, Thoroughbred, and Welsh Pony) that represent major groups of worldwide equine genetic diversity (Petersen et al., 2013).

### 2.2 Mapping and Variant Calling

Standard fastq quality control and trimming were performed using Fastqc 0.11.8 and Trimmomatic 0.38, respectively. Raw reads were aligned to the EquCab 3.0 reference horse assembly (Kalbfleisch et al., 2018) and variants identified using a modified version of the genome analysis toolkit best practices (Van der Auwera et al., 2013), modified to allow for joint variant calling by GATK haplotype caller (McKenna et al., 2010) and BCFtools mpileup (Li H. et al., 2009). In brief, reads were mapped to the EquCab 3.0 reference genome using Burrows-Wheeler Aligner (BWA) (Li and Durbin 2009). PCR duplicates were detected and removed using Picard tools (https://broadinstitute.github.io/picard/) version 2.18.27 and then indel realignment was performed using the Genome Analysis Toolkit (GATK) version 4.1.0.0 indel realigner and base-quality score recalibration was performed (DePristo et al., 2011). Genome variant call format (genome VCF) files were produced for each individual horse, and then group variant calling was performed using GATK haplotype caller version 4.1.0.0 (McKenna et al., 2010) and BCFtools mpileup version 1.9 (Li H. et al., 2009) using default settings. Hard-filtering was performed using the GATK best practice guidelines (Van der Auwera et al., 2013). To maximize the specificity of the variants, the intersection of the variants across both callers was obtained using GATK “SelectVariants” and was used for downstream analysis (Van der Auwera et al., 2013).

### 2.3 Variant Analyses

Descriptive statistics for both variant callers and the intersection were created using BCFtools (Li 2011). Missingness for each horse and each variant site was calculated using VCFtools. The transition to transversion (TsTv) and heterozygous to non-reference homozygous (hetNRhom) ratios were calculated for each variant caller, across the population and by breed using BCFtools (Li 2011) and Python. Predicted functional effect for each variant in the intersect file was determined using SnpEff (Cingolani et al., 2012b) with a custom dictionary based on the RefSeq version of EquCab 3.0 (Schaefer et al., 2017). High, moderate, and low impact variants were extracted using SnpSift (Cingolani et al., 2012a) and Python was used to manipulate VCF files. Python and BCFtools (Li H. et al., 2009) were used to manipulate output files.

### 2.4 Breed Differences Between Variants

Variants that were considered rare (<3%) or common (>10%) were extracted from each breed VCF file using Python and BCFtools (Li H. et al., 2009). Variants that were rare in one breed and common in another, rare/common or common/rare in the breed/population, or uniquely present in one breed were selected for investigation. BCFtools view was used to extract variants that were only present in homozygous states, or were present in all genotyped individuals. Python was to extract variant details.

### 2.5 Regions of the Genome With High or Low Genetic Variation

BCFtools stats was used on 10 kb regions across the genome. R was used to calculate the average genetic variation and to find regions with high (more than twice the average variation) and low (less than half the average variation) levels of genetic variation. BCFtools view was used to extract these regions from the intersect. Python was then used to extract variant details and additional analysis performed with R.

### 2.6 Statistical Analyses

A Kruskal Wallis test and linear regression were used to determine if there were breed differences. The nonlinear relationship between depth of coverage and the number of variants identified was determined using R. Due to the association between depth of coverage and the number of variants detected, estimated marginal means (EMMEANS) (Lenth 2018) were calculated, with depth of coverage included in the regression models. T tests were used to compare variant types and allele frequencies between coding and non-coding variants, and to compare constraint metric scores between high and low variation regions. A chi-square test was used to compare the variant impact between the high and low variation regions. Confidence intervals (95%) were calculated for each breed. All statistical analyses were performed using R. Significance was set at *p* < 0.05.

## 3 Results

### 3.1 Variant Discovery

WGS of 534 horses across 46 different breeds (Supplementary Table S2), was performed on Illumina platforms (Supplementary Table S1). This sample set included DNA from a minimum of 15 horses in each of 10 target breeds (Arabian, Belgian, Clydesdale, Icelandic Horse, Morgan, Quarter Horse, Shetland Pony, Standardbred, Thoroughbred, and Welsh Pony) that represent major breed clusters in the horse (Petersen et al., 2013), which were sequenced to a target depth of coverage (DOC) of 10X. Raw reads were mapped, quality control was performed, and variants were identified using a modified version of the genome analysis toolkit best practices pipeline (Van der Auwera et al., 2013) (see Section 2). 155,201,208,820 total reads from the 534 horses mapped uniquely to the EquCab 3.0 reference genome (Kalbfleisch et al., 2018). Mean and median read length, uniquely mapped paired reads, and depth of coverage, and ranges for these values are provided in Table 1.

**TABLE 1:** Median, mean, and range of summary statistics of the mapping pipeline derived from WGS data from 534 horses.

	Median	Mean	Range
Read length (bp)	99.6	114.5	73.9–234.2
Uniquely mapped paired end reads	240,563,458	290,638,968	17,030,804–1,536,494,934
Depth of coverage (X)	9.2	11.5	1.4–46.7

**FIGURE 1:**
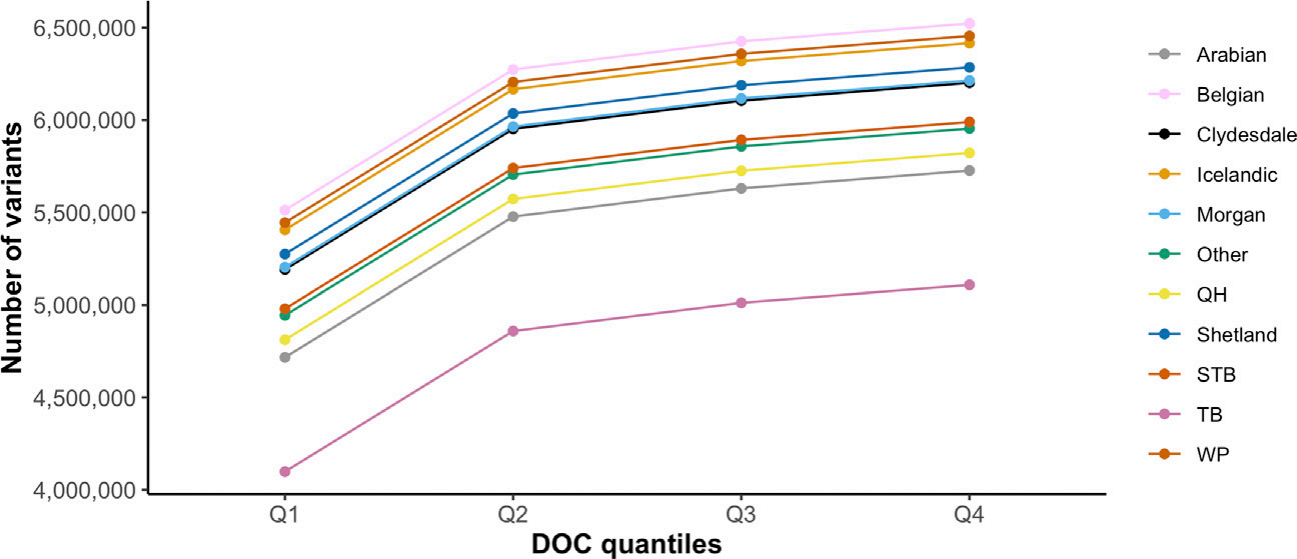
Distribution of the average number of variants identified for each breed by DOC quantiles (Q1 = 1.43–7.14 X, Q2 = 7.15–9.16 X, Q3 = 9.17–14.6 X, Q4 = 14.7–46.7 X). The colored lines represent the 10 target horse breeds [Arabian, Belgian, Clydesdale, Icelandic horse, Morgan horse, Quarter Horse (QH), Shetland, Standardbred (STB), Thoroughbred (TB), and Welsh Pony (WP)] and the remaining horse breeds (Other).

**FIGURE 2:**
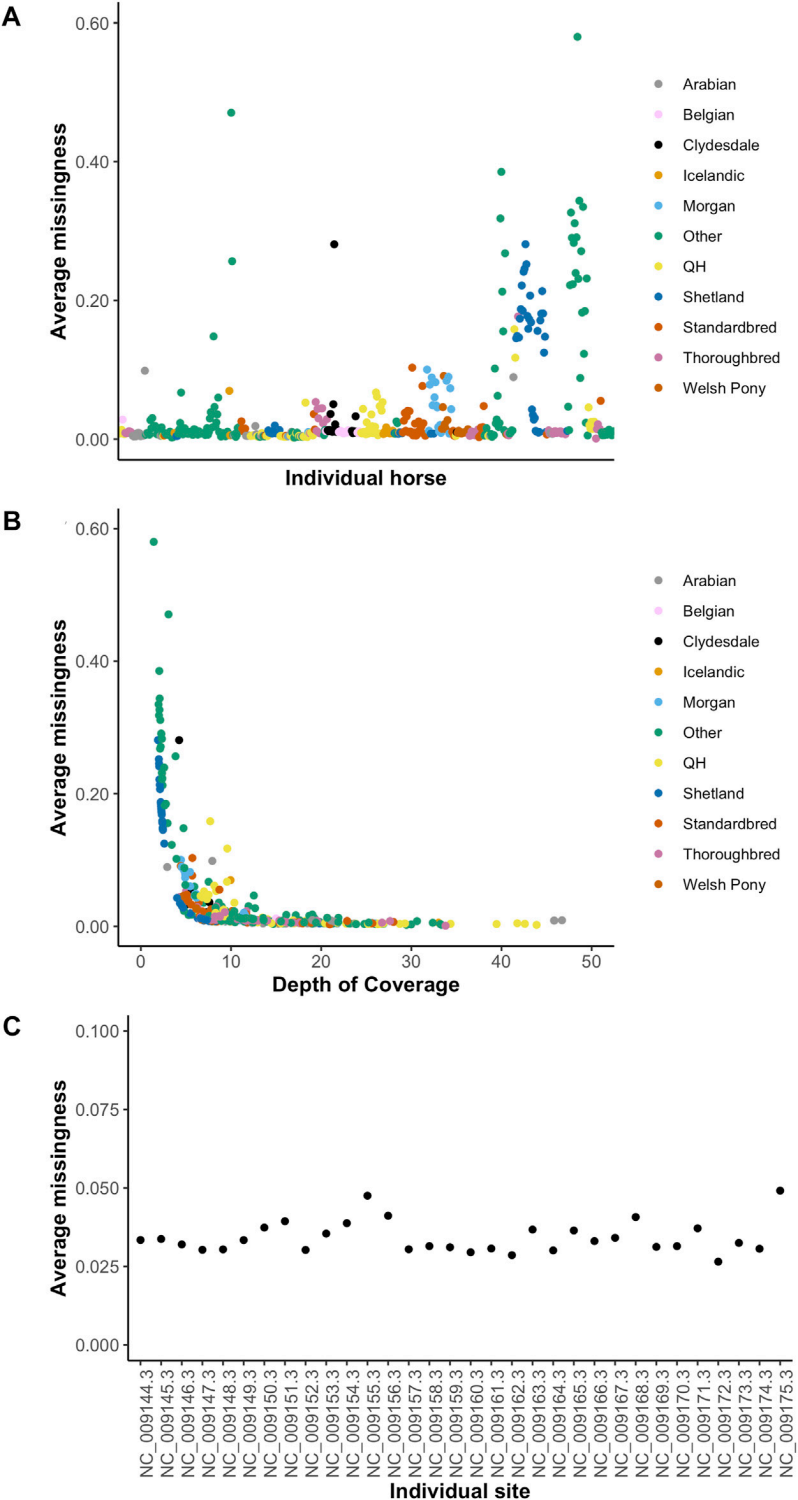
Missingness by individual **(A)**, by depth of coverage **(B)** and by chromosome **(C)**. The 10 target breeds and other breeds are represented in colors shown in the figure legend in Figures 2A,B.

GATK Haplotype Caller and BCFtools identified 42,900,494 and 33,395,275 variants, respectively. To increase specificity of the identified variants, the intersect of both variant callers [31,140,769 variants (29,038,030 SNPs and 2,102,379 indels)] was used for downstream analysis (Table 2). On average, there were 2.27 variants (range 0.88–3.12) per kb of sequence. The mean number of heterozygous sites per individual per kb was 1.54 (range 0.56–2.63). There was a significant non-linear association between the number of variants identified and the depth of coverage [DOC (correlation estimate 0.62, *p*_adjusted_ 0.009)]. The distribution of variants by DOC was similar for each breed (Figure 1), therefore, estimated marginal means (EMMEANs) (Lenth 2018) accounting for DOC were used for further analyses. The median (range) degree of missingness for each individual horse was 0.01 (0.001–0.58) and for each variant site was 0.026 (0.000–0.998) (Figure 2). Missingness by individual was moderately negatively correlated with DOC: correlation −0.45, 95% confidence interval −0.511 to −0.38 and *p* < 0.001 (Figure 2).

**TABLE 2:** Number of variants, TsTv, and HetNRhom ratio from 534 WGS identified by each variant caller [GATK Haplotype Caller (HC) and BCFtools (BT), and the union and intersection of the variant callers].

Variant Caller	Variants	SNPs	INDELs	MA sites	MA SNP sites	TsTv ratio	HetNRhom ratio
HC	42,900,494	38,205,867	4,694,627	2,974,935	2,127,391	1.87	2.48
BT	33,395,275	30,642,613	2,752,662	783,956	295,703	2.08	2.18
Union	45,154,996	439,810,450	5,344,546	3,298,397	2,178,474	1.54	2.17
Intersect	31,140,769	29,038,030	2,102,379	1,547,737	990,223	1.94	2.24

MA, multiallelic; TsTv, transition/transversion; HetNRhom, heterozygous-non-reference homozygous.

**FIGURE 3:**
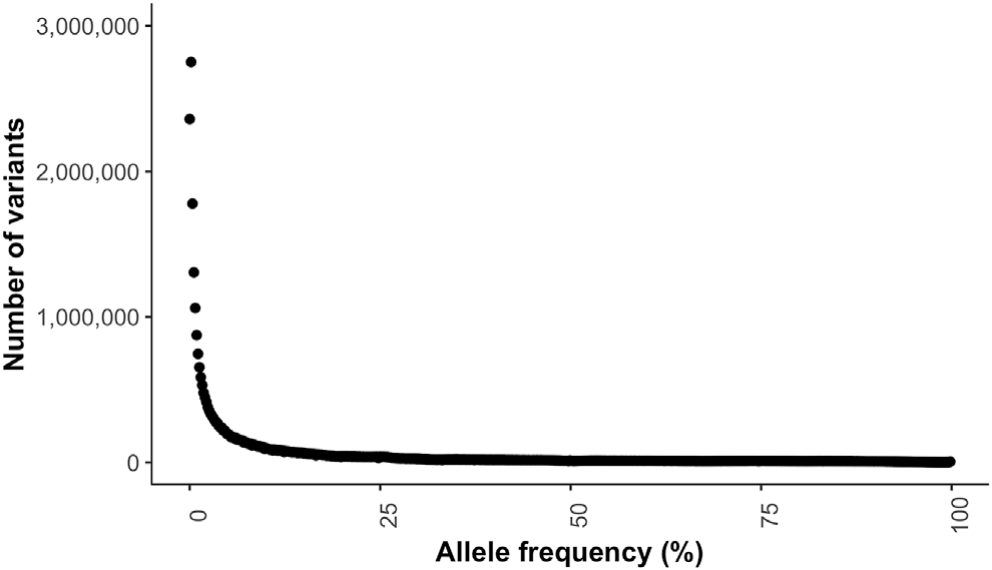
Minor allele frequency distribution of the variants.

The transition to transversion (TsTv) ratio and the heterozygous to non-reference homozygous (hetNRhom) ratios from the variant callers intersect were 1.94 and 2.24, respectively (Table 2). There were significant but marginal breed differences in the TsTv and hetNRhom ratios (*p* < 0.001), with the highest TsTv ratio in Shetlands (1.95) and the lowest in Thoroughbreds (1.92), The same was true with the hetNRhom ratio, which was the highest in Thoroughbreds (3.21) and the lowest in Clydesdales (1.48) (Table 3). The majority (57%) of variants had a minor allele frequency (MAF) < 5%, Figure 3), with the mean MAF being 13.2% (0.09–100%). In total, there were 2,481,075 (2,447,610 SNPs and 33,465 indels) singleton variants.

**TABLE 3:** Estimated marginal mean of the transition to transversion (TsTv) and heterozygous to non-reference homozygous (hetNRhom) ratios accounting for depth of coverage.

Breed	TsTv ratio	hetNRhom ratio
Arabian	1.93	1.53
Belgian	1.94	2.02
Clydesdale	1.93	1.68
Icelandic	1.94	1.89
Morgan	1.94	2.26
Quarter Horse	1.93	2.26
Shetland	1.95	2.42
Standardbred	1.93	2.03
Thoroughbred	1.92	3.19
Welsh Pony	1.94	2.27

Each individual horse had on average 5,580,202 variants (5,099,978 SNPs and 480,224 indels), with on average 1,805,127 in homozygous and 3,775,075 in heterozygous states (Table 4). There were also breed-specific differences in variant number and homozygous variant number per individual (Table 4). The EMMEAN variants per individual, accounting for DOC, was lowest in Thoroughbreds (5,000,516) and highest in Belgians (6,100,544). The EMMEAN homozygous variants per individual, accounting for DOC, was also lowest in Thoroughbreds (1,225,441) and highest in Belgians (2,325,470).

**TABLE 4:** EMMEANs for number of variants within breeds accounting for DOC, standard error (SE), and 95% confidence intervals, with breed and DOC as predictor variables.

Breed	Variants included	EMMEAN	SE	Lower confidence interval	Upper confidence interval
Average	All	5,580,202	33,642	5,514,039	5,646,364
Arabian	All	5,587,952	54,820	5,480,322	5,695,582
Belgian	All	6,100,544	67,559	5,967,903	6,233,186
Clydesdale	All	5,977,299	69,379	5,841,084	6,113,515
Icelandic	All	6,093,155	72,659	5,950,500	6,235,810
Morgan	All	5,602,287	67,541	5,469,681	5,734,893
Quarter Horse	All	5,473,401	39,427	5,395,992	5,550,810
Shetland	All	5,645,668	44,377	5,558,541	5,732,794
Standardbred	All	5,623,603	44,981	5,535,289	5,711,917
Thoroughbred	All	5,000,516	43,061	4,915,972	5,085,060
Welsh Pony	All	5,836,729	67,893	5,703,431	5,970,028
Average	Homozygous	1,805,127	10,931	1,783,628	1,826,626
Arabian	Homozygous	1,812,878	54,820	1,705,248	1,920,508
Belgian	Homozygous	2,325,470	67,559	2,192,828	2,458,111
Clydesdale	Homozygous	2,202,225	69,379	2,066,010	2,338,440
Icelandic	Homozygous	2,318,081	72,659	2,175,426	2,460,736
Morgan	Homozygous	1,827,212	67,541	1,694,607	1,959,818
Quarter Horse	Homozygous	1,698,327	39,427	1,620,918	1,775,735
Shetland	Homozygous	1,870,593	44,377	1,783,466	1,957,720
Standardbred	Homozygous	1,848,529	44,981	1,760,215	1,936,843
Thoroughbred	Homozygous	1,225,441	43,061	1,140,897	1,309,985
Welsh Pony	Homozygous	2,061,655	67,893	1,928,356	2,194,953

The top half of the table provides the EMMEAN for the total number of variants per individual (All) and the bottom half of the table provides the total number of homozygous variants present in each individual.

The EMMEAN variant number per breed was significantly correlated with one estimation of effective population size across breeds (Petersen et al., 2013) (*p* = 0.02, Pearson’s correlation = 0.83, 95% confidence interval 0.22–0.97), but not a more recent estimate of effective population size across breeds using a higher marker density (Beeson et al., 2019) (*p* = 0.54, Pearson’s correlation = 0.26, 95% confidence interval −0.54–0.82). The EMMEAN homozygous variant number per breed was not significantly correlated with either estimate (Petersen et al., 2013; Beeson et al., 2019) of effective population size (*p* = 0.07, Pearson’s correlation = 0.72, 95% confidence interval −0.08–0.95 and *p* = 1, Pearson’s correlation = −0.001, 95% confidence interval −0.70–0.70, respectively).

### 3.2 Variants Shared Across Breeds

In total, 27,719,724 variants (25,386,978 SNPs and 2,332,746 indels) were shared by at least two breeds (shared variants, Supplementary Table S3). Only 2% (637,610) of these variants were genic (within 5,000 bp of a gene). Genic variants included 8,427 high (4,934 SNPs and 3,493 indels), 199,243 moderate (195,630 SNPs and 3,613 indels), and 429,940 low (427,436 SNPs and 2,504 indels) impact variants with 6,697 predicted to cause loss of function (LOF). Genic variants were within or near 10,013 individual genes. The mean shared variant MAF was 0.07. The mean shared variants per breed was 13,802,105 (range 11,800,825 in Clydesdales to 15,724,634 in Quarter Horses).

### 3.3 Variants With Large Allele Frequency Discrepancies

10,633,492 variants (121,900 genic) were considered rare (MAF <3%) in one breed and common (MAF >10%) in at least one other breed (Supplementary Table S4). Each breed had on average 1,216,800 variants that had large allele frequency discrepancies with another breed. The fewest number of variant discrepancies were between the Quarter Horse and Thoroughbred (728,459 variants) and the highest number of variant discrepancies were between the Thoroughbred and the Shetland (2,028,658 variants).

4,876,293 variants (190,653 genic) were rare (MAF <3%) in one breed and common (MAF >10%, mean MAF 0.15) in the remainder of the study population (excluding individuals of that breed) (breed-specific rare variants, Supplementary Table S4). Each genic variant was present in or close to at least one of 10,361 genes. The mean number of breed-specific rare variants was 475,458. The fewest number of variant discrepancies was between the Quarter Horse and the population (8,248 variants) and the highest number of variant discrepancies was between the Thoroughbred and the population (913,739).

3,563,454 variants (62,803 coding) were common (MAF >10%) in one breed and rare (MAF <3%, mean MAF 0.02) in the remaining population (excluding individuals of that breed) (breed-specific common variants, Supplementary Table S4). On average, each of these variants was present in 1.13 breeds (range 1–5) and each genic variant was shared by 1.11 breeds (range 1–4). Genic variants were present in or close to at least one of 10,163 genes. On average, each breed had 444,464 variants that were common in the breed and rare in the population. The fewest number of variant discrepancies was between the Thoroughbred and the population (115,241 variants) and the highest number of variant discrepancies was between the Icelandic horse and the population (745,623).

### 3.4 Variants With No Homozygotes Present

2,889 variants (2,586 SNPs, 303 indels) were only present in a heterozygous state, with no homozygotes identified. Twenty-six of these variants were present within 14 different genes. 12/14 of these genes are uncharacterized or equine specific transcripts or olfactory related genes. Six were predicted to be high impact (four were predicted to be LOF variants), 10 were predicted to be moderate impact, and 10 were predicted to be low impact variants (Supplementary Table S5). The allele frequency was marginally but significantly different (*p* = 0.003) between non-genic (0.498) and genic (0.499) variants.

### 3.5 Variants Present in all Horses

114,733 variants (103,414 SNPs, 11,319 indels) were present in all 534 horses. Of these variants, 1,426 were present in genic regions of 504 genes. These variants were predicted to have a high (170 variants), moderate (644 variants) and low (612 variants) impact on phenotype, with 145 predicted to be LOF variants. Of these variants, 9,756 (9,351 SNPs, 405 indels) were homozygous in all horses. Ninety-two of these were present in genic regions affecting 58 genes. Eight were predicted to be high impact (four were predicted to be LOF), 46 were predicted to be moderate impact, and 38 were predicted to be low impact variants.

### 3.6 Regions of the Genome With High or Low Genetic Variation

The variant caller intersect file was first split by chromosome and then into 10,000 bp windows (240,910 regions in total) to determine the average number of variants per 10 Kb region across the genome. Regions with more than two times or less than half of the average number of variants were classified as regions of high or low variability, respectively. Each 10 Kb region carried on average 122 variants (range 0–3,143 variants), consisting of 114 SNPs (range 0–3,075 SNPs) and 8 indels (range 0–121). There were 6,341 regions with more than double the mean variant number, including, on average 414 variants (396 SNPs and 18 indels) with a TsTv ratio of 1.76. There were 17,791 regions with less than half the average number of variants, including, on average 35 variants (32 SNPs and three indels) with a TsTv ratio of 1.59.

**FIGURE 4:**
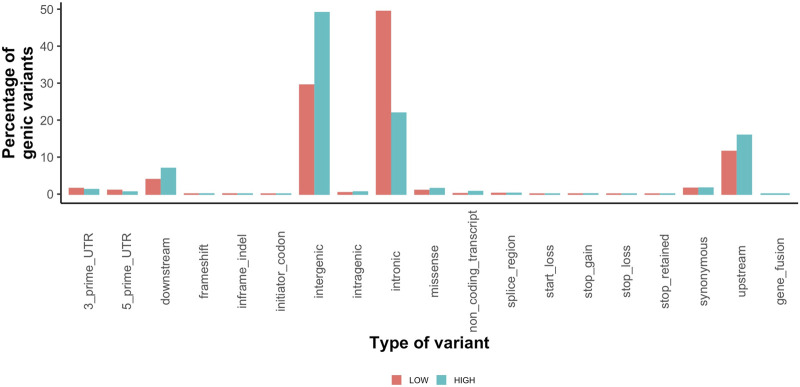
Percentage of coding variants for each type of variant called by SnpEff for low variation regions (orange) and high variation regions (teal).

Highly variable regions contained 2,625,382 variants (Supplementary Table S6) with a mean MAF of 17% (range 0.01–100%). The most common variant types in the high variability regions were intergenic (1,287,507) and intronic (574,441) (Figure 4). The low variability regions contained 20,777 variants with a mean MAF of 11% (range 0.01–100%). The most common variant types in the low variation regions were intronic (306,569) and intergenic (182,937) variants (Figure 4). The variant impact was significantly different between regions of high and low variation [*p* < 0.001 (Table 5)].

**TABLE 5:** Impact of variants identified in high and low variation regions.

	High	Moderate	Low	Modifier
**High variation region**	2061	39,260	48,234	2,535,827
**Low variation region**	303	6,293	11,521	602,660

The allele frequency of the variants in the low variability regions was significantly less than the allele frequency of variants in high variability regions (*p* < 0.001, 95% confidence interval: 0.06–0.06). For the genes with haploinsufficiency scores available, the mean score was lower (*p* < 0.001, 95% confidence interval: −0.15 to −0.10) in genes containing variants in the high variability regions (0.23) compared to genes containing variants in the low variability regions (0.33). The predicted loss of function tolerance score was not significantly different (*p* = 0.13, 95% confidence interval: −0.07–0.01) between genes containing variants in the high variability regions (0.33) compared to genes containing variants in the low variability regions (0.37).

## 4 Discussion

This report comprises the first large-scale catalog of genetic variation developed for the horse, a species with potential as a translational model for many athletic phenotypes. We used WGS of 534 horses to determine overall genetic variation in the general equine population as well as 10 individual breeds, report variants with population and breed MAF discrepancies, identify variants with no homozygotes as well as variants that are present in all individuals, and in addition identify genomic regions with high or low genetic variation.

The significant association between the number of variants identified and the depth of coverage was unsurprising, as it has long been recognized that deeper coverage improves variant calling accuracy (Li R. et al., 2009). The nonlinear association between depth of coverage and number of variants identified is particularly relevant to future genetic studies, as there is minimal to no gain in the numbers of variants detected by increasing depth of coverage over 10X.

We elected to use the intersect of two commonly used callers based on evidence from previous work showing that specificity of identified variants could be improved (Field et al., 2015). This method identified 29,882,273 variants, which is higher than the 25,800,000 variants previously identified in a smaller cohort of 88 horses using only GATK haplotype caller (Jagannathan et al., 2019b). This difference is likely due to the increased number of horses and inclusion of more genetically distinct breeds in our study, increasing our ability to identify rare variants down to a MAF across breeds of 0.0009 compared with 0.0057 in the earlier study. Additionally, we use the intersect of two variant callers (GATK HaplotypeCaller and BCFtools) to improve specificity of variants identified in the previous publication (Jagannathan et al., 2019b). While this likely would have led to a reduced sensitivity, we were still able to identify an additional 4,082,273 variants.

The 1.54 heterozygous variants per kb of sequence is similar to that reported in cattle (1.44 per kb) (Daetwyler et al., 2014), but is higher than reported in the Yoruba (1.03 per kb) and European human populations (0.68 per kb) (Genomes Project et al., 2010). The cause of this higher variation in the horse is likely related to the heterogeneity of this population, which included 46 different breeds, compared to three cattle breeds (Daetwyler et al., 2014) and only a single population in the human studies (Genomes Project et al., 2010). Given the limited genetic diversity (Petersen et al., 2013) of the horse compared with human populations this is still somewhat somewhat surprising. However, a recent study of effective population size in the horse suggests that several breeds have larger effective population sizes (Beeson et al., 2019) than reported in human populations (Tenesa et al., 2007), and we would therefore, expect to see increased heterozygosity. Another reason for the increased number of heterozygous variants in horses is likely to be related to errors in the reference genome which, unlike the human reference genome that is based on multiple individuals, is based on a single horse (Kalbfleisch et al., 2018). In the original paper from the 1,000 human genomes consortium, it was concluded that a site where every individual was homozygous for an allele not present in the reference genome was a reference genome error. This accounted for ∼1 error per 30 kb of sequence. In this study, we identified 114,733 variants that were present in every individual in this population, which are presumed to be related to an error in the reference genome or a true rare variant that is present in the individual horse sequenced for the reference genome. The length of the RefSeq equine reference genome is 2,506.95 MB, therefore, we would expect to see ∼1.37 errors per 30 kb of sequence, which may partially explain the increased number of variants in horses compared with humans.

Unsurprisingly, the degree of missingness per individual was negatively correlated with depth of coverage. This was not a linear correlation however, and beyond 10X coverage, there was minimal improvement in the degree of missingness, suggesting that 10X coverage is a reasonable target for population scale sequencing projects. The average missingness per individual varied greatly. Most of the horses with missingness >0.20 were horses that were not included in the breed analysis owing to being in the other breed category. Six of the Shetland ponies had missingness >0.20 and these were ponies with a targeted depth of coverage of ∼ 6X. The degree of missingness across the 32 autosomes and X chromosomes also varied, with the highest degree of missingness on chromosomes 12 and X. This may be related to larger uncharacterized regions on the X chromosome compared to most autosomes.

The TsTv and hetNRhom ratios are frequently utilized for quality control of sequencing data (Guo et al., 2013). The TsTv ratio here was similar (1.94) to reports of the expected TsTv ratio from genome sequencing data in humans of ∼2 (Genomes Project et al., 2010), which is a good indicator of SNP quality (Guo et al., 2013; Wang et al., 2015). The slight reduction in our study compared with human studies is likely related to decreased genetic diversity and smaller effective population sizes in horses (Petersen et al., 2013) compared with humans, as well as a lower quality reference genome (Wade et al., 2009). The TsTv ratio varies across the genome, but in humans does not vary based on ancestry (Wang et al., 2015). In the 10 target breeds, we did find that the TsTv ratio varied significantly but marginally by ancestry. This may be related to the varying depth of sequence coverage, which was not uniform across breeds. The hetNRhom ratio (2.24) was higher than the expected value of 2 based on Hardy-Weinberg equilibrium (Guo et al., 2013) and there were breed differences. This is consistent with human (Wang et al., 2015) and canine (Jagannathan et al., 2019a) reports that the hetNRhom ratio varies by ancestry. In humans, the highest median hetNRhom ratio was 2.0 in African populations with the lowest ratio of 1.4 in Asian populations; none of the populations investigated had a median hetNRhom ratio >2.0 (Wang et al., 2015). However, at least one dog breed had hetNRhom ratio of 3.3 in the catalog of canine genetic variation (Jagannathan et al., 2019a). This is likely related to the increased levels of inbreeding in horses compared with most human populations.

The variant totals differed by breed, which is consistent with reports in different cattle breeds (Daetwyler et al., 2014) and regional human populations (Genomes Project et al., 2010). Previously, this has been related to effective population size (Daetwyler et al., 2014), and we did see a significant association (*p* < 0.0001) between the number of variants identified in each of the 10 target breeds and a report of effective population size using 54K SNP array data (Petersen et al., 2013). However, this association was not seen with a more recent estimate of effective population size that used imputed genome-wide SNP data (Beeson et al., 2019). This may partially be related to different breeds studied, as the Beeson et al. (2019) paper did not include effective population size estimates for the Shetland and Clydesdale breeds. Additionally, we are not accounting for the degree of relatedness between the breeds studied and the reference genome. In this study, the Thoroughbred (which is the EquCab3 reference genome breed) has the fewest variants compared to the other breeds, consistent with both the reference genome being from a Thoroughbred and having the smallest effective population size (1,784) in Beeson et al. (Beeson et al., 2019). However, the Quarter Horse, which has the largest population size (6,516) (Beeson et al., 2019), but is more related to the Thoroughbred (Petersen et al., 2013) than other breeds, has a number of variants that is closer to the median, consistent with its close relatedness to the Thoroughbred (Petersen et al., 2013), but inconsistent with the large effective population size (Beeson et al., 2019). This would suggest, that while the number of variants does vary by breed, as seen in different human regional populations, the relationship between the horse breed and the reference genome appears to have had an effect in this population.

A large number of variants were shared by at least one other breed, which is not surprising given the close relatedness of the breeds investigated (Petersen et al., 2013). However, there were also multiple variants with large allele frequency discrepancies between breeds. We defined a minor allele frequency <3% as rare and >10% as common due to limitations in the number of horses in each breed investigated, rather than values used in most human studies (<0.5% for rare variants and >5% for common (Bomba et al., 2017)). With the 534 horses here our power to detect variants present at a minor allele frequency of 3 and 0.5% in the population is 1.00 and 0.93, respectively. However, it is important to note that for the breed analysis with the least number of horses in the Clydesdales (19) our power to detect these allele frequencies is only 0.44 and 0.01, respectively. To have a power greater than 0.8 to detect all rare variants <3% or <0.5% allele frequency within a breed we would need to sequence 55 and 325 horses within that breed, respectively. We therefore, had 80% power to detect the rare variants (minor allele frequency 3%) in Quarter Horses, Shetlands and Thoroughbreds. The number of variants with marked population discrepancies was quite high, with ∼35% of variants considered rare in one breed and common in another. The reason for large allele frequency differences between populations is thought to be related to genetic drift (Hofer et al., 2009) in humans. However, given that different horse breeds have been selectively bred for different traits (Avila et al., 2018), it is also likely that selection at least partially accounts for some of the variants with large allele frequency discrepancies between breeds. Only ∼5% of variants were unique to a single breed, with about 10% of these being in coding regions. The ∼5% of unique variants in horses is also lower than seen in cattle where ∼31% of variants are unique to a single breed (Daetwyler et al., 2014).

Variants with no homozygotes were explored to determine if there were variants present in the general population that could be embryonic lethal in homozygous form. 2,888/2,889 of these variants were present at a minor allele frequency in the general population greater than would be expected for a homozygous lethal disease (MAF > 0.10). The one variant that was rare had a MAF of 0.03 and was an intergenic in frame deletion. Using the rules of Hardy-Weinberg equilibrium we would need to sequence 1,111 horses to identify just one homozygote, therefore using this dataset alone, we cannot determine if this variant is embryonic lethal. Given that 96% of the variants with no homozygotes have an allele frequency around 0.50, it is highly unlikely that these variants are embryonic lethal; rather it is possible that these regions are related to mapping errors due to the presence of paralogs or pseudogenes, or the presence of structural variants. This is supported by the fact that all but two of the genes containing these variants were uncharacterized or equine only transcripts or olfactory receptor genes.

Identifying regions of the genome with high or low variation in the general population is critically important for the investigation of possible disease-causing variants, as regions with high genetic variability are less likely to contain disease-causing variants for fully penetrant Mendelian diseases (Karczewski et al., 2019). However, our analysis unexpectantly found that genes in high variability regions had lower haploinsufficiency scores, suggesting that damaging variants are less well tolerated (Huang et al., 2010) than for genes present in the low variability regions. This is likely due to the large number of genes without haploinsufficiency scores (70% of high variation region genes and 23% of low variation region genes). 65% of genes in high and 18% of genes in low variation regions had the “LOC” designation that are genes unique to the horse or are only predicted to be equivalent to a human gene, and therefore would not be included in human databases of variant constraint. This would suggest that as expected, genes in the low variability regions are more similar to human genes than genes in the high variability regions.

Overall, this is the first large-scale catalog of genetic variation in the domestic horse and will be highly useful for evaluation of background genetic variation in any future genetic study. This catalog has paved the way for future investigation of the regions of the genome that are shared or have marked MAF discrepancies across breeds. Regions with marked discrepancies between breeds, or between a breed and the population, can then be interrogated to further look for signatures of selection both across breeds, as well as within breeds. Additionally, further investigation into which of the variants that are present in all individuals are related to poor genome annotation or instead are true variants in the reference genome is needed and will lead to improvement of the horse reference genome in the future. By improving knowledge of the poorly annotated regions of the horse genome we will be able to correct these for future versions of the reference genome. This will have benefits for future phenotype-causing variant identification studies. Given the relatively unique utility of the horse as a model for human athletic related traits (Hill et al., 2010; Rooney et al., 2018) and diseases (Ward et al., 2004; McCue et al., 2008; McIlwraith et al., 2012; McCoy et al., 2013; Norton et al., 2016), an improved ability to identify phenotype-causing variants in the horse may shed light on analogous genetic diseases in humans. This is especially true for complex phenotypes such as athleticism, osteoarthritis (McIlwraith et al., 2012), and exertional rhabdomyolysis (Norton et al., 2016) where the limited genetic diversity in the horse (Petersen et al., 2013) may further accelerate our ability to identify the true phenotype-causing variants. This improvement in the reference genome combined with an improved understanding of the background genetic variation (Yang et al., 2014; Farwell et al., 2015; Ellingford et al., 2016; Hartmannová et al., 2016; Känsäkoski et al., 2016; Kojima et al., 2016; Noll et al., 2016; Smedley et al., 2016; Dolzhenko et al., 2017; Eldomery et al., 2017; Schneider et al., 2017) in the horse should vastly increase the identification of phenotype-causing variants for important equine diseases.

## Data Availability

The variant datasets presented in this study can be found online at: https://www.ncbi.nlm.nih.gov/bioproject/PRJEB47918.
