# Supplementary material for: Genetic Variation and the Distribution of Variant Types in the Horse
Source: Front Genet. 2021 Dec 2;12:758366. doi: 10.3389/fgene.2021.758366 (PMC8676274; doi:10.3389/fgene.2021.758366)
Supplement: Supplementary file 1 [file DataSheet1.docx]

| **Supplementary table 1.** Sequencing platform for the whole genome sequence FASTQs | |
| --- | --- |
| **Instrument type** | **Number of horses** |
| Illumina HiSeq 2500 | 99 |
| Illumina HiSeq 3000 | 6 |
| Illumina HiSeq | 161 |
| Illumina NovaSeq | 109 |
| Unknown platform | 159 |

| **Supplementary table 2.** Breed and number of WGS represented in the database of genetic variants. | | |
| --- | --- | --- |
| **Breed** | **Number of WGS** | **Mean (range) depth of coverage (X)** |
| Arab* | 35 | 18.8 (2.9 - 46.7) |
| Belgian* | 20 | 8.3 (6.3 - 15.0) |
| British Warmblood | 2 | 14.7 (14.5 - 14.9) |
| Clydesdale* | 19 | 7.6 (4.2 - 10.1) |
| Coldblood | 3 | 11.9 (11.2 - 12.7) |
| Connemara | 4 | 5.5 (5.3 - 5.6) |
| Cross breed | 5 | 14.6 (3.1 - 33.2) |
| Curly Trotter | 2 | 12.5 (12.4 - 12.6) |
| Duelmener | 1 | 12.4 (12.4 - 12.4) |
| Franchese Montagne | 30 | 12.9 (7.0 - 19.8) |
| French Trotter | 10 | 8.8 (8.4 - 8.7) |
| Friesian | 2 | 8.55 (8.4 - 8.7) |
| German warmblood | 9 | 17.6 (10.7 - 33.3) |
| Haflinger | 8 | 17.1 (8.2 - 21.3) |
| Hanovarian | 7 | 14.0 (8.9 - 23.9) |
| Holsteiner | 3 | 13.6 (10.7 - 17.4) |
| Icelandic* | 17 | 10.0 (6.5 - 33.0) |
| Italian Trotter | 1 | 32.4 (32.4 - 32.4) |
| Jeju pony | 21 | 3.8 (1.4 - 20.7) |
| KWPN (Dutch warmblood) | 1 | 9.4 (9.4 - 9.4) |
| Lipizzaner | 4 | 18.5 (17.2 - 19.2) |
| Mangalarga Marchador Horse | 1 | 8.9 (8.9 - 8.9) |
| Miniature horse | 4 | 7.6 (4.8 - 11.2) |
| Mongolian | 9 | 5.8 (2.0 - 10.4) |
| Morgan* | 20 | 8.4 (4.4 - 25.9) |
| Native Mondolian Chakouyi Horse | 1 | 11.7 (11.7 - 11.7) |
| Norwegian Fjord | 1 | 7.5 (7.5 - 7.5) |
| Oldenberg | 2 | 15.3 (14.0 - 16.7) |
| Percheron | 3 | 12.6 (9.2 - 14.5) |
| Pony of the Americas | 1 | 14.7 (14.7 - 14.7) |
| Quarter Horse* | 76 | 15.5 (6.6 - 43.9) |
| Saxon-Thuringian Heavy Warmblood | 1 | 13.6 (13.6 - 13.6) |
| Shetland* | 55 | 5.6 (1.9 - 21.8) |
| Sorraia | 3 | 11.5 (11.5 - 11.6) |
| Sports Horse | 2 | 26.4 (26.0 - 26.9) |
| Standardbred* | 50 | 11.6 (4.8 - 28.0) |
| Swiss Warmblood | 3 | 9.9 (7.6 - 12.4) |
| Tennessee Walking Horse | 4 | 19.0 (8.2 - 29.5) |
| Thoroughbred* | 54 | 11.4 (2.3 - 33.8) |
| Trakenher | 2 | 14.7 (9.9 - 19.4) |
| UK Warmblood | 1 | 15.5 (15.5 - 15.5) |
| Unknown | 6 | 14.0 (2.1 - 30.6) |
| Warmblood | 2 | 21.1 (19.0 - 23.2) |
| Welsh Pony* | 20 | 7.1 (4.4 - 9.0) |
| Westphalian | 2 | 15.9 (13.6 - 18.11) |
| Yakut | 9 | 14.6 (10.7 - 25.3) |
| **Total** | **534** | **11.5 (1.5 - 46.7)** |
| *10 target breeds that represent major breed groups of genetic diversity (Petersen et al., 2013) | | |

| **Supplementary table 3.** Number of shared variants by breed. | | | | | | | | | | |
| --- | --- | --- | --- | --- | --- | --- | --- | --- | --- | --- |
| **Breed** | **Arabian** | **Belgian** | **Clydesdale** | **Icelandic** | **Morgan** | **QH** | **Shetland** | **STB** | **TB** | **WP** |
| **Arabian** | NA | 13,266,138 | 11,413,193 | 12,621,192 | 13,710,064 | 15,892,702 | 14,495,737 | 14,025,123 | 13,522,598 | 14,179,465 |
| **Belgian** | 13,266,138 | NA | 11,863,752 | 12,900,205 | 13,533,820 | 15,832,579 | 14,641,793 | 13,605,265 | 12,691,836 | 14,137,987 |
| **Clydesdale** | 11,413,193 | 11,863,752 | NA | 11,111,495 | 11,558,844 | 13,086,177 | 12,370,031 | 11,712,533 | 10,999,186 | 12,092,210 |
| **Icelandic** | 12,621,192 | 12,900,205 | 11,111,495 | NA | 12,824,239 | 14,784,764 | 14,594,466 | 13,173,289 | 12,216,344 | 13,367,713 |
| **Morgan** | 13,710,064 | 13,533,820 | 11,558,844 | 12,824,239 | NA | 16,076,174 | 14,728,208 | 14,571,424 | 13,265,484 | 14,271,661 |
| **QH** | 15,892,702 | 15,832,579 | 13,086,177 | 14,784,764 | 16,076,174 | NA | 17,421,381 | 16,415,926 | 15,371,772 | 16,640,233 |
| **Shetland** | 14,495,737 | 14,641,793 | 12,370,031 | 14,594,466 | 14,728,208 | 17,421,381 | NA | 14,913,521 | 14,079,604 | 15,442,271 |
| **STB** | 14,025,123 | 13,605,265 | 11,712,533 | 13,173,289 | 14,571,424 | 16,415,926 | 14,913,521 | NA | 13,743,224 | 14,381,451 |
| **TB** | 13,522,598 | 12,691,836 | 10,999,186 | 12,216,344 | 13,265,484 | 15,371,772 | 14,079,604 | 13,743,224 | NA | 13,547,636 |
| **WP** | 14,179,465 | 14,137,987 | 12,092,210 | 13,367,713 | 14,271,661 | 16,640,233 | 15,442,271 | 14,381,451 | 13,547,636 | NA |

| **Supplementary table 4.** Variants with marked allele frequency differences (i.e. rare [MAF <3%] in one group and common [MAF >10%] in the other) between breeds or between breeds and the general population excluding that breed. | | | | | | |
| --- | --- | --- | --- | --- | --- | --- |
|  | **Total number of variants** | | **Number of coding variants** | | | **Number of LOF variants** |
|  | **SNPs** | **indels** | **High impact** | **Moderate impact** | **Low impact** |  |
| **Rare in one breed and common in another breed** | 10,633,492 | | 121,900 | | | 1,985 |
|  | 9,724,996 | 908,496 | 2,650 | 47,474 | 71,776 |  |
| **Rare in one breed and common in the population** | 4,876,293 | | 190,653 | | | 1,262 |
|  | 4,555,601 | 320,692 | 1,634 | 58,838 | 130,181 |  |
| **Common in one breed and rare in the population** | 3,563,454 | | 62,803 | | | 656 |
|  | 3,299,971 | 263,483 | 882 | 19,966 | 41,955 |  |

| **Supplementary table 5.** Predicted variant impact, location and consequence, and number of variants with no homozygotes. Genes containing these variants are listed for the variants of high, moderate, and low predicted impacts. | | | | | |
| --- | --- | --- | --- | --- | --- |
| **Predicted variant impact** | **Variant location/consequence** | | **Number** | **Genes affected** | **Gene details** |
| High | Frameshift | | 1 | CUNH12orf40 | Uncharacterized protein – C12orf40 homolog |
|  | Splice region | Frameshift | 2 | GFM1 | G elongation factor mitochondrial 1 – Role in regulation of normal mitochondrial function and in different disease states attributed to mitochondrial dysfunction isn’t known. |
|  |  |  |  | OR5A1P | Olfactory Receptor Family 5 Subfamily A Member 1 - Olfaction |
|  |  | Acceptor/donor | 2 | LOC102149590 | Uncharacterized protein, equine – PANTHER protein class – ATP-binding cassette transporter |
|  |  |  |  | OR5D18DP | Olfactory receptor family 5 subfamily D member 18D, equine pseudogene |
|  | Nonsense | | 1 | THSD1 | Thrombospondin type 1 domain containing 1 – involved in the complement pathway. |
| Moderate | Missense | | 10 | LOC100629999 | rho GTPase-activating protein 20, equine – neurite outgrowth. |
|  |  |  |  | LOC102149709 | Disks large-associated protein 5, equine |
|  |  |  |  | LOC102149904 (n = 2) | Neuroblastoma breakpoint family member 6, equine |
|  |  |  |  | LOC106781177 | Ral guanine nucleotide dissociation stimulator-like, equine |
|  |  |  |  | OR2AJ23 | Olfactory receptor family 2 subfamily AJ member 3, equine |
|  |  |  |  | OR4D11 | Olfactory receptor family 4 subfamily D member 11 |
|  |  |  |  | OR4D9FP (n = 2) | Olfactory receptor family 4 subfamily D member 9F, pseudogene |
|  |  |  |  | OR4D9I | Olfactory receptor family 4 subfamily D member 9I, equine |
| Low | 5’ UTR premature start codon | | 4 | LOC102149515 (n = 2) | Uncharacterized gene, equine |
|  |  |  |  | OR4D9I | Olfactory receptor family 4 subfamily D member 9I, equine |
|  |  |  |  | PPFIA1 | Protein tyrosine phosphatase receptor type F polypeptide interacting protein alpha 1 – member of the liprin family, important for axon guidance and mammary gland development. |
|  | Synonymous | | 6 | CUNH12orf40 | Uncharacterized protein – C12orf40 homolog |
|  |  |  |  | LOC100629999 | rho GTPase-activating protein 20, equine – neurite outgrowth. |
|  |  |  |  | LOC102149709 (n = 2) | Disks large-associated protein 5, equine |
|  |  |  |  | OR4D11 | Olfactory receptor family 4 subfamily D member 11 |
|  |  |  |  | OR4D9FP | Olfactory receptor family 4 subfamily D member 9F, pseudogene, equine |
| Modifier | 3’ UTR | | 11 |  |  |
|  | 5’ UTR | | 20 |  |  |
|  | Downstream/upstream gene | | 584 |  |  |
|  | Intergenic | | 1,731 |  |  |
|  | Intragenic | | 43 |  |  |
|  | Intron | | 441 |  |  |
|  | Noncoding transcript | | 33 |  |  |

| **Supplementary table 6.** Variants present in 10 kb windows of high (>2 times the average number of variants per window) or low (<0.5 times the average number of variants per window) variation in the general population | | | | | | |
| --- | --- | --- | --- | --- | --- | --- |
|  | **Number of regions** | **Total number of variants** | | **Total number of coding variants** | | |
|  |  | **SNPs** | **indels** | **High impact** | **Moderate impact** | **Low impact** |
| **High variation regions** | 6,341 | 2,625,382 | | 89,555 | | |
|  |  | 2,509,755 | 115,627 | 2,061 | 39,260 | 48,234 |
| **Low variation regions** | 17,791 | 620,777 | | 18,117 | | |
|  |  | 569,603 | 51,174 | 303 | 6,293 | 11,521 |
